# Acute Coronary Syndrome: Unravelling the Biology to Identify New Therapies

**DOI:** 10.3390/cells11244136

**Published:** 2022-12-19

**Authors:** Bradley Tucker, Sanjay Patel

**Affiliations:** 1 Royal Prince Alfred Hospital, Camperdown, Sydney 2050, Australia; 2 Sydney Medical School, University of Sydney, Camperdown, Sydney 2050, Australia; 3 Faculty of Medicine, University of New South Wales, Kensington, Sydney 2033, Australia; 4 Heart Research Institute, Newtown, Sydney 2042, Australia

Acute coronary syndrome (ACS) encompasses a spectrum of presentations including unstable angina, non-ST elevation myocardial infarction (NSTEMI) and ST-elevation myocardial infarction (STEMI). There are multiple aetiologies for ACS, including atherothrombosis, coronary artery dissection, coronary artery spasm, microvascular dysfunction and coronary embolism [1]. Of these, atherothrombosis is by far the most common pathology resulting in ACS. In this issue, Khandkar et al. [2] reviewed the most recent evidence regarding the pathophysiology of atherothrombosis and discussed the role of intracoronary imaging to determine the underlying cause of thrombosis and to ultimately guide ACS management. Additionally, Vaidya et al. [3] reviewed the crucial role of coronary microcirculation in ACS and its potential as a therapeutic target. Complications of atherosclerotic disease, including ACS, ischaemic non-cardioembolic stroke and peripheral artery disease, are the leading causes of morbidity and mortality worldwide. Despite current primary prevention strategies including lifestyle optimisation, glycaemic control, blood pressure lowering and hyperlipidaemia management, in 2018, there were 1.2 million unique hospitalisations for ACS in the United States alone [4]. For those who have a non-fatal event, one in three will have recurrence within 5 years [5]. Even for those on maximum lipid lower therapy who achieve an LDL cholesterol level < 1 mmol/L, there is a 10% risk of a major adverse cardiovascular event (MACE) in the following 2 years [6]. Further evidence has demonstrated that typical cardiovascular risk factors (hypertension, hypercholesterolaemia, diabetes mellitus and smoking) do not adequately capture the total cardiovascular risk, particularly in contemporary populations. Vernon and colleagues demonstrated that the prevalence of STEMI patients with none of the four aforementioned risk factors has increased almost 3-fold from 11% in 2006 to 27% in 2014. Importantly, several observational studies have demonstrated that this population without traditional risk factors have worse short-term outcomes [7,8,9]. These data demonstrate that current primary and secondary prevention strategies are insufficient and that alternative pathogenic mechanisms remain ill-defined and untreated. In order to develop new strategies to manage the diverse population of ACS patients, we must better understand its pathobiology. 

The role of inflammation in atherosclerotic disease has gained much attention in recent times, largely due to the publication of several cardiovascular outcome trials, namely CANTOS [10], COLCOT [11] and LoDoCo2 [12]. These trials confirmed a causal role of inflammation in atherosclerotic cardiovascular disease (ASCVD) and marked it as a viable therapeutic target. In fact, the anti-atherogenic effect of some well-known medications, including statins and sodium-glucose cotransporter 2 (SGLT2) inhibitors, is partially mediated via their anti-inflammatory properties [13,14]. The role of SGLT2 inhibitors and their potential mechanism in ASCVD is discussed in-depth by Barraclough et al. [13] in this issue. The anti-inflammatory agents canakinumab and colchicine have been shown to attenuate atherosclerosis-associated inflammation, thereby reducing cardiovascular events. However, this reduction in cardiovascular risk comes at the cost of compromised immunity and ultimately no difference in overall mortality [15]. This area of research has reached an important crossroads where one of two things must happen for patients to see this reduction in cardiovascular events translated into a meaningful reduction in all-cause mortality. Either future anti-inflammatory agents need to target more downstream processes in the inflammatory cascade to limit off-target immunosuppression, or patient selection needs to be refined. The former involves greater basic science research into the inflammatory pathways involved in ASCVD and the development of highly specific antibodies or small-molecule inhibitors. The latter, patient selection, is being addressed by the ongoing COLCARDIO-ACS study (ACTRN12616000400460)—a randomised trial of colchicine versus placebo in 3000 ACS patients. This study is recruiting patients with a recent ACS and elevated high sensitivity C-reactive protein ((hsCRP) >2 mg/L 4—52 weeks post-ACS) to determine whether post-ACS inflammatory status modifies the treatment effect of colchicine. It is hypothesised that by selecting a population with an elevated baseline, hsCRP participants in this trial will have a higher event rate and derive greater benefit from anti-inflammatory therapy compared to those in previous trials. The prevalence of an elevated hsCRP (>2 mg/L) post-ACS is estimated to be 50% [16]. Longitudinal data have demonstrated that a persistently elevated hsCRP is a strong independent predictor of future MACE and all-cause mortality. In fact, when compared to those with an hsCRP < 2 mg/L, those with an hsCRP persistently > 2 mg/L have a two-fold higher risk of MACE and a three-fold higher risk of all-cause mortality in the one year following percutaneous coronary intervention [17]. Although hsCRP has proven to be a robust predictor of future MACE, it is limited by its lack of specificity for atherosclerosis-associated inflammation. More specific biomarkers of coronary inflammation have been developed, including imaging of pericoronary adipose tissue (as a surrogate marker of vascular inflammation) and positron emission tomography. As discussed in this issue, these biomarkers can accurately reflect localised coronary inflammation, thereby identifying vulnerable plaque and predicting future MACE with greater specificity than systemic inflammatory markers [18,19,20]. As these modalities become more widely available, they will aid in risk stratification and prognostication and may serve as predictive biomarkers for the response to anti-inflammatory therapy in the post-ACS setting. 

The residual risk of ASCVD not captured by traditional risk factors cannot solely be explained by inflammation. ACS is an acute manifestation of a complex chronic disease influenced by both environmental and genetic factors. To fully understand the pathophysiology of ASCVD, we must investigate multiple biological levels including metabolomics, genomics, proteomics, lipidomics, transcriptomics, and immunophenotyping. The Australian BioHEART study is well placed to produce such multi-omic data. In this issue, Vernon and colleagues [21] performed liquid chromatography mass spectrometry on plasma samples from 1002 patients in the BioHEART-CT study. This study identifies metabolic biomarkers of specific coronary artery disease (CAD) phenotypes and demonstrates a novel association of the nucleotide metabolic pathway with CAD—a finding that warrants further investigation at the basic science level. Research included in this Special Issue also identified PCSK7 as a possible pathogenic factor in ACS. PCSK7 is an enzyme responsible for the regulation of triglycerides and HDL cholesterol levels. In a large Mexican population, Vargas-Alarcón et al. [22] identified three single-nucleotide polymorphisms (rs508487 T/C, rs236911 C/A, and rs236918 C/G) associated with an increased risk of ACS—likely via their HDL-cholesterol-lowering and/or triglyceride-elevating effect.

In summary, this Special Issue highlights recent advancements in the understanding of ACS pathobiology. The reviews in this issue highlight the multiple pathogenic mechanisms involved in ACS and identify many potential therapeutic targets. The original research presented here aids our understanding of this complex disease—ultimately bringing us closer to unravelling its biology and developing targeted therapeutics.

## References

[1] Libby P. (2013). Mechanisms of Acute Coronary Syndromes and Their Implications for Therapy. New Engl. J. Med..

[2] Khandkar C., Madhavan M.V., Weaver J.C., Celermajer D.S., Karimi Galougahi K. (2021). Atherothrombosis in Acute Coronary Syndromes—From Mechanistic Insights to Targeted Therapies. Cells.

[3] Vaidya K., Tucker B., Patel S., Ng M.K.C. (2021). Acute Coronary Syndromes (ACS)—Unravelling Biology to Identify New Therapies—The Microcirculation as a Frontier for New Therapies in ACS. Cells.

[4] Tsao C.W., Aday A.W., Almarzooq Z.I., Alonso A., Beaton A.Z., Bittencourt M.S., Boehme A.K., Buxton A.E., Carson A.P., Commodore-Mensah Y. (2022). Heart Disease and Stroke Statistics-2022 Update: A Report From the American Heart Association. Circulation.

[5] Steen D.L., Khan I., Andrade K., Koumas A., Giugliano R.P. (2022). Event Rates and Risk Factors for Recurrent Cardiovascular Events and Mortality in a Contemporary Post Acute Coronary Syndrome Population Representing 239 234 Patients During 2005 to 2018 in the United States. J. Am. Heart Assoc..

[6] Sabatine M.S., Giugliano R.P., Keech A.C., Honarpour N., Wiviott S.D., Murphy S.A., Kuder J.F., Wang H., Liu T., Wasserman S.M. (2017). Evolocumab and Clinical Outcomes in Patients with Cardiovascular Disease. N. Engl. J. Med..

[7] Kong G., Chin Y.H., Chong B., Goh R.S.J., Lim O.Z.H., Ng C.H., Muthiah M., Foo R., Vernon S.T., Loh P.H. (2023). Higher mortality in acute coronary syndrome patients without standard modifiable risk factors: Results from a global meta-analysis of 1,285,722 patients. Int. J. Cardiol..

[8] Vernon S.T., Coffey S., Bhindi R., Soo Hoo S.Y., Nelson G.I., Ward M.R., Hansen P.S., Asrress K.N., Chow C.K., Celermajer D.S. (2017). Increasing proportion of ST elevation myocardial infarction patients with coronary atherosclerosis poorly explained by standard modifiable risk factors. Eur. J. Prev. Cardiol..

[9] Vernon S.T., Coffey S., D’Souza M., Chow C.K., Kilian J., Hyun K., Shaw J.A., Adams M., Roberts-Thomson P., Brieger D. (2019). ST-Segment-Elevation Myocardial Infarction (STEMI) Patients Without Standard Modifiable Cardiovascular Risk Factors-How Common Are They, and What Are Their Outcomes?. J. Am. Heart Assoc..

[10] Ridker P.M., Everett B.M., Thuren T., MacFadyen J.G., Chang W.H., Ballantyne C., Fonseca F., Nicolau J., Koenig W., Anker S.D. (2017). Antiinflammatory Therapy with Canakinumab for Atherosclerotic Disease. N. Engl. J. Med..

[11] Tardif J.-C., Kouz S., Waters D.D., Bertrand O.F., Diaz R., Maggioni A.P., Pinto F.J., Ibrahim R., Gamra H., Kiwan G.S. (2019). Efficacy and Safety of Low-Dose Colchicine after Myocardial Infarction. N. Engl. J. Med..

[12] Nidorf S.M., Fiolet A.T.L., Mosterd A., Eikelboom J.W., Schut A., Opstal T.S.J., The S.H.K., Xu X.-F., Ireland M.A., Lenderink T. (2020). Colchicine in Patients with Chronic Coronary Disease. N. Engl. J. Med..

[13] Barraclough J.Y., Patel S., Yu J., Neal B., Arnott C. (2021). The Role of Sodium Glucose Cotransporter-2 Inhibitors in Atherosclerotic Cardiovascular Disease: A Narrative Review of Potential Mechanisms. Cells.

[14] Diamantis E., Kyriakos G., Quiles-Sanchez L.V., Farmaki P., Troupis T. (2017). The Anti-Inflammatory Effects of Statins on Coronary Artery Disease: An Updated Review of the Literature. Curr. Cardiol. Rev..

[15] Fiolet A.T.L., Opstal T.S.J., Mosterd A., Eikelboom J.W., Jolly S.S., Keech A.C., Kelly P., Tong D.C., Layland J., Nidorf S.M. (2021). Efficacy and safety of low-dose colchicine in patients with coronary disease: A systematic review and meta-analysis of randomized trials. Eur. Heart J..

[16] Ridker P.M. (2017). How Common Is Residual Inflammatory Risk?. Circ. Res..

[17] Kalkman D.N., Aquino M., Claessen B.E., Baber U., Guedeney P., Sorrentino S., Vogel B., de Winter R.J., Sweeny J., Kovacic J.C. (2018). Residual inflammatory risk and the impact on clinical outcomes in patients after percutaneous coronary interventions. Eur. Heart J..

[18] Bartlett B., Ludewick H.P., Lee S., Verma S., Francis R.J., Dwivedi G. (2021). Imaging Inflammation in Patients and Animals: Focus on PET Imaging the Vulnerable Plaque. Cells.

[19] Yuvaraj J., Cheng K., Lin A., Psaltis P.J., Nicholls S.J., Wong D.T.L. (2021). The Emerging Role of CT-Based Imaging in Adipose Tissue and Coronary Inflammation. Cells.

[20] Yuvaraj J., Lin A., Nerlekar N., Munnur R.K., Cameron J.D., Dey D., Nicholls S.J., Wong D.T.L. (2021). Pericoronary Adipose Tissue Attenuation Is Associated with High-Risk Plaque and Subsequent Acute Coronary Syndrome in Patients with Stable Coronary Artery Disease. Cells.

[21] Vernon S.T., Tang O., Kim T., Chan A.S., Kott K.A., Park J., Hansen T., Koay Y.C., Grieve S.M., O’Sullivan J.F. (2021). Metabolic Signatures in Coronary Artery Disease: Results from the BioHEART-CT Study. Cells.

[22] Vargas-Alarcón G., Pérez-Méndez O., González-Pacheco H., Ramírez-Bello J., Posadas-Sánchez R., Escobedo G., Fragoso J.M. (2021). The rs508487, rs236911, and rs236918 Genetic Variants of the Proprotein Convertase Subtilisin–Kexin Type 7 (*PCSK7*) Gene Are Associated with Acute Coronary Syndrome and with Plasma Concentrations of HDL-Cholesterol and Triglycerides. Cells.

